# Significant association of RANKL polymorphisms with bone metastasis among patients with breast cancer

**DOI:** 10.3389/fonc.2026.1788455

**Published:** 2026-04-07

**Authors:** Haoyu Lian, Siyuan Zhou, Huanyang He, Qian Liu, Jinmin Zhao

**Affiliations:** 1 Guangxi Key Laboratory of Regenerative Medicine, Orthopaedic Department, The First Affiliated Hospital of Guangxi Medical University, Nanning, China; 2 Collaborative Innovation Centre of Regenerative Medicine and Medical BioResource Development and Application Co-constructed by the Province and Ministry, Life Sciences Institute, Guangxi Medical University, Nanning, China; 3 Orthopedics, Ezhou Central Hospital, Ezhou, China

**Keywords:** bone metastasis, breast cancer, polymorphisms, *RANKL*, single nucleotide polymorphism

## Abstract

**Objective:**

The purpose of this study was to reveal the genetic correlation of *RANKL* polymorphisms with bone metastasis in breast cancer patients.

**Methods:**

In this study, 139 bone metastasis patients and 152 no metastasis were included as the case and control groups. Real-time polymerase chain reaction (PCR) and allelic discrimination method were respectively applied for the genotyping of rs7325635 and rs2277438. Polymorphism genotype and allele frequencies were compared by χ^2^ test between the two groups. The risk of bone metastasis development caused by *RANKL* genetic variants was evaluated by odds ratio (OR) with 95% confidence interval (95%CI). The linkage of two polymorphisms was examined by Haploview. Binary and multivariate logistic analyses were used to optimize the results.

**Results:**

Rs2277438 GG genotype and G allele frequencies were significantly higher in bone metastasis patients than that in no-metastasis patients (*P* < 0.05); they were significantly correlated with the increased risk of bone metastasis occurrence (GG: OR = 2.065, 95%CI=1.104-3.863; G: OR = 1.486, 95%CI=1.068-2.068). Compared with the G–G haplotype, the A–A haplotype was found to significantly reduce the risk of bone metastasis in breast cancer patients (OR = 0.647, 95%CI=0.436-0.959). The multivariate logistic analysis indicated that family history, Ki67, and rs2277438 were positively correlated with bone metastasis, but menopausal state, clinical staging, and rs7325635 were negatively correlated with bone metastasis in breast cancer.

**Conclusion:**

The *RANKL* rs2277438 variant may be a potential genetic susceptibility factor associated with bone metastasis risk in breast cancer patients, though further functional validation is warranted. Rs7325635 was not independently associated with bone metastasis. The linkage disequilibrium between these two polymorphisms and their combined haplotype effect may play a role in bone metastasis susceptibility.

## 1 Introduction

Breast cancer is one of the most frequent cancers among women now (1). According to the report of IARC (International Agency for Research on Cancer) in 2018 based on 185 countries, 2.3 million new cases of breast cancer are discovered and account for 11.7% of all people (2, 3). Breast cancer is also the leading cause of cancer-related deaths, with 685,000 deaths all over the world in 2020 (4). Bone metastasis is the most common distant metastatic site and takes up over 70% of metastatic breast cancer cases (5, 6). A 5-year survival rate in metastatic breast cancer is less than 30%, even with adjuvant chemotherapy (7). The bone metastasis occurrence of breast cancer is a complex process and affected by a mass of factors including genetic and environmental factors (8–10). Especially, genetic factors may exert the vital function when breast cancer metastasizes to the bones (11). However, existing studies are not enough to expound the pathogenesis of bone metastasis in breast cancer.

RANKL (receptor activator of nuclear factor-kappa B ligand), also known as TNFSF11 (TNF superfamily member 11), is encoded by the *RANKL* gene. It belongs to the tumor necrosis factor (TNF) cytokine family and is a ligand for osteoprotegerin, and it functions as a key factor for osteoclast differentiation and activation (12, 13). It is firstly found to participate in the bone systems (14). *RANKL* is usually a key regulator for osteoclast differentiation, survival, and activity (15). It is reported to activate canonical osteoclastogenic signaling pathways and induce osteoclastogenic transcriptional and epigenetic programs in osteoclastogenesis through binding to its receptor RANK (16–18). Except for bone metabolism, RANKL also serves as the key regulator for immune tolerance, antitumor immunity, and mammary gland biology (19). Previously, *RANKL* had been reported to participate in the development, progression, and treatment of breast cancer (20). In the recent year, it is also found to be involved in bone metastasis of breast cancer patients (21). The role of *RANKL* genetic variants in bone metastasis of breast cancer is also paid attention to by scholars nowadays.

In the current research, we discussed the association of *RANKL* polymorphisms with the risk of bone metastasis in patients with breast cancer. Two common functional polymorphic sites in RANKL were selected, namely, rs7325635 and rs2277438. These two SNPs were chosen for the following reasons: (1) both are located in the intron region of RANKL and have been previously reported to be associated with bone-related phenotypes. Rs7325635 has been significantly correlated with hip bone loss in postmenopausal women (33), and rs2277438 has shown a significant correlation with bone diseases such as osteoporosis (34). (2) A linkage disequilibrium between these two SNPs has been reported in previous studies, suggesting their potential combined effect on bone metabolism. (3) Both SNPs have relatively high minor allele frequencies in East Asian populations according to the 1000 Genomes Project database, making them suitable for association studies with adequate statistical power in the Chinese Han population. The linkage analysis of *RANKL* polymorphisms was also conducted in the occurrence of bone metastasis. Multivariate logistic analysis of clinical features and *RANKL* polymorphisms in bone metastasis occurrence of breast cancer patients was also carried out.

## 2 Materials and methods

### 2.1 Study population description

The sample size was estimated using PASS 15.0 software prior to the study. Based on the minor allele frequency of the rs2277438 G allele in the East Asian population (approximately 0.38, according to the 1000 Genomes Project), with a significance level of 0.05, a statistical power of 80%, and an expected odds ratio of 2.0 for the risk genotype, a minimum of 130 cases and 130 controls were required. A *post hoc* power analysis confirmed that the current sample size (139 cases and 152 controls) provided approximately 82% power to detect an OR of 2.0 at the 0.05 significance level for the GG genotype comparison.

We collected 139 breast cancer patients with bone metastasis and 152 breast cancer patients without metastasis or recurrence from The First Affiliated Hospital of Guangxi Medical University between January 2023 and September 2024 as the case and control groups, respectively. Included criteria of study subjects were as follows: they were diagnosed as breast cancer patients through histopathological or cytological examination, or focal biopsy. Bone lesion was examined by X-ray, CT, or MRI examination and confirmed as bone metastasis patients through at least two senior doctors. The excluded criteria were as follows: bone metabolic disease, bisphosphonates, hormones, and calcium treatment were used or received radiotherapy within 4 weeks prior to the screening; pregnant or lactating women; had other serious diseases such as diabetes. The controls were frequency-matched with the cases in age.

All subjects were Han people of northern China without blood relationship to each other. This study design had obtained the support of the Ethics Committee of The First Affiliated Hospital of Guangxi Medical University. The execution of this experiment was in accordance with the World Medical Association Declaration of Helsinki. Written informed consents were acquired from every participator before the sampling.

### 2.2 DNA extraction

3–5 ml peripheral venous blood was drawn from every subject after their fasting period for at least 8 h and placed into a blood collection tube, rocked up and down promptly for preventing the coagulation. Genomic DNA was extracted from peripheral blood samples using the Maxwell 16 Blood DNA Purification Kit (Promega Corporation) and silica membranes (Qiagen), according to the producers’ instructions.

### 2.3 The genotyping of RANKL polymorphisms

The quality of extracted genomic DNA was assessed by a NanoDrop 2000 spectrophotometer (Thermo Fisher Scientific), and samples with A260/A280 ratios between 1.8 and 2.0 were considered acceptable for genotyping. *RANKL* rs7325635 genetic variants were genotyped using the LightSNiP assay (TIB MOLBIOL, Berlin, Germany) on a LightCycler 480 real-time PCR device (Roche Diagnostics, Mannheim, Germany). The PCR cycling conditions were as follows: initial denaturation at 95 °C for 10 min, followed by 45 cycles of 95 °C for 10 s, 60 °C for 10 s, and 72 °C for 15 s, with a final melting curve analysis. The genotyping of rs2277438 polymorphism was performed by allelic discrimination using the 5′ nuclease PCR assay with TaqMan SNP Genotyping Assay (Applied Biosystems). The detector used was an ABI Prism 7200 sequence detection platform (Perkin Elmer). The PCR conditions were as follows: 95 °C for 10 min, then 40 cycles of 95 °C for 15 s and 60 °C for 1 min. For quality control, approximately 10% of samples were randomly selected for duplicate genotyping, and the concordance rate was 100%. The overall genotyping call rate was >98% for both SNPs. Negative controls (no-template controls) were included in each genotyping plate.

### 2.4 Statistical analysis

The genotype and allele frequencies of *RANKL* polymorphisms were assessed by direct counting. The clinical traits of breast cancer patients with or without bone metastasis were compared by Student’s t-test (continuous variables) and χ^2^ test (categorical variables). The Hardy–Weinberg equilibrium (HWE) was assessed for genotype distributions in the control group for each polymorphism using the goodness-of-fit χ^2^ test, comparing observed genotype frequencies with expected frequencies calculated from allele frequencies. A P value > 0.05 indicated that the genotype distribution was consistent with HWE. χ^2^ test was also adopted for comparing the genotype and allele frequencies of *RANKL* polymorphisms between the two groups. Odds ratio (OR) and 95% confidence interval (95%CI) were calculated to estimate the risk intensity of bone metastasis occurrence caused by *RANKL* genetic variants in breast cancer patients. The results were adjusted by clinical traits of the subjects through logistic regression analysis. The linkage of polymorphisms was also examined by Haploview. All data management was conducted by PASW Statistics 18.0 software (SPSS, Inc., Chicago, IL, USA). A multivariate logistic analysis was also carried out. Since only two SNPs were tested in this study, Bonferroni correction was applied to adjust for multiple comparisons. The corrected significance threshold was set at P < 0.025 (0.05/2). *P* < 0.05 was considered as the statistically significant difference.

## 3 Results

### 3.1 The clinical information of bone metastasis and no metastasis in breast cancer patients

We firstly investigated and analyzed the demographics and clinical features of breast cancer patients with and without bone metastasis. The average age of bone metastasis patients and no-metastasis patients were 54.97 ± 12.73 and 53.72 ± 13.73 years; the difference was not significant between the two groups (*P* = 0.423). In our study population, obesity was not significantly related to bone metastasis in breast cancer patients (*P* = 0.078). Family history of breast cancer and menopausal state were obviously correlated with bone metastasis of breast cancer patients (*P* < 0.05), but not pathological type. Moreover, bone metastasis (*P* = 0.035) easily developed in breast cancer patients with high clinical staging (stages III-IV). However, HER2 (human epidermal growth factor receptor 2), PR (progesterone receptor), and ER (estrogen receptor) statuses were found to be not correlated with bone metastasis of breast cancer patients (*P*>0.05). Molecular subtyping had significant influence on bone metastasis (*P* = 0.020). We also discovered that breast cancer on the left side was more likely to develop bone metastasis (*P* = 0.020), and Ki67 was also an important indicator for bone metastasis in breast cancer patients (*P* < 0.001). The detailed data and results are displayed in **Table 1**.

**Table 1:** Demographics, clinical features of breast cancer patients in different groups.

Characteristics	Bone metastasis (n=139)	No metastasis (n=152)	P
Age (year)	54.97 ± 12.73	53.72 ± 13.73	0.423
Obesity			0.078
Yes	71 (51.08)	60 (39.47)	
No	68 (48.92)	92 (60.53)	
Family history of breast cancer			**0.011**
Yes	31 (22.30)	17 (11.18)	
No	108 (77.70)	135 (88.82)	
Menopausal state			**0.025**
Menopausal	92 (66.19)	81 (53.29)	
Premenopausal	47 (33.81)	71 (46.71)	
Pathological type			0.080
Invasive type	96 (69.06)	90 (59.21)	
Non-invasive type	43 (30.94)	62 (40.79)	
Clinical staging			**0.035**
Stages I-II	42 (30.22)	64 (42.11)	
Stages III-IV	97 (69.78)	88 (57.89)	
Tumor size			0.202
≤ 3 cm	44 (31.65)	59 (38.82)	
> 3 cm	95 (68.35)	93 (61.18)	
HER2 status			0.451
Positive	57 (41.01)	69 (45.39)	
Negative	82 (58.99)	83 (54.61)	
PR status			0.968
Positive	68 (48.92)	74 (48.68)	
Negative	71 (51.08)	78 (51.32)	
ER status			0.831
Positive	76 (54.68)	85 (55.92)	
Negative	63 (45.32)	67 (44.08)	
Molecular subtyping			**0.022**
Luminal A	36 (25.90)	52 (34.21)	
Luminal B	37 (26.62)	40 (26.32)	
HER2 overexpression	45 (32.37)	27 (17.76)	
TNBC	21 (15.11)	33 (21.71)	
Location			**0.020**
Left side	91 (65.47)	79 (51.97)	
Right side	48 (34.53)	73 (48.03)	
Ki67			**<0.001**
< 14%	11 (7.91)	47 (30.92)	
> 14%	128 (92.09)	105 (69.08)	

HER2, human epidermal growth factor receptor 2; PR, progesterone receptor; ER, estrogen receptor; TNBC, triple negative breast cancer.

Bold values indicate statistical significance (P < 0.05).

### 3.2 The association of RANKL polymorphisms with bone metastasis in breast cancer patients

The genotype and allele frequencies of *RANKL* rs7325635 and rs2277438 polymorphisms in the case and control groups are shown in **Table 2**. First of all, the genotype distributions of rs7325635 and rs2277438 polymorphisms were both consistent with HWE (*P*>0.05, **Table 2**). For rs7325635, three genotypes GG, AG, and AA were identified; their frequencies were 56.12%, 35.97%, and 7.91% in the bone metastasis group, and 47.37%, 41.45%, and 11.18% in the no-metastasis group, respectively. However, there was no significant difference in genotypes of rs7325635 between the two groups (*P*>0.05). Similarly, its G and A allele frequencies were not significantly different between the bone metastasis group and no-metastasis group (*P* = 0.111).

**Table 2:** The genotype and allele distributions of *RANKL* polymorphisms between breast cancer patients with and without bone metastasis.

Genotype/ allele	Case (n=139, %)	Control (n=152, %)	*P*	OR (95%CI)	*P* ^a^	OR^a^ (95%CI)
rs7325635						
GG	78 (56.12)	72 (47.37)	–	Ref.		
AG	50 (35.97)	63 (41.45)	0.213	0.733 (0.449-1.196)	0.208	0.730 (0.446-1.192)
AA	11 (7.91)	17 (11.18)	0.217	0.597 (0.262-1.361)	0.205	0.587 (0.257-1.339)
*P*_HWE_		0.569				
G	206 (74.10)	207 (68.09)	–	Ref.		
A	72 (25.90)	97 (31.91)	0.111	0.746 (0.520-1.070)		
rs2277438						
AA	46 (33.09)	63 (41.45)	–	Ref.		
AG	54 (38.85)	63 (41.45)	0.550	1.174 (0.694-1.986)	0.546	1.176 (0.695-1.992)
GG	39 (28.06)	26 (17.10)	**0.023**	**2.054 (1.100-3.838)**	**0.023**	**2.065 (1.104-3.863)**
*P*_HWE_		0.143				
A	146 (52.52)	189 (62.17)	–	Ref.		
G	132 (47.48)	115 (37.83)	**0.019**	**1.486 (1.068-2.068)**		

HWE, Hardy–Weinberg equilibrium; OR, odds ratio; 95%CI, 95% confidence interval. a: The values of *P* and OR were adjusted by clinical features of subjects.

Bold values indicate statistical significance (P < 0.05).

In rs2277438, AA, AG, and GG genotype frequencies were 33.09%, 38.85%, and 28.06% in the bone metastasis group and 41.45%, 41.45%, and 17.10% in the no-metastasis group, respectively. Its GG genotype frequency was significantly higher in bone metastasis patients than that in no-metastasis patients (*P* = 0.023), which remained significant after Bonferroni correction (corrected threshold P < 0.025), but not AG genotype (*P* = 0.550); its GG genotype was obviously related to the increased risk of bone metastasis in breast cancer patients (OR = 2.054, 95%CI=1.100-3.838). Even if it was adjusted by age, the association still existed between the two (adjusted OR = 2.065, 95%CI=1.104-3.863). Furthermore, compared to the A allele, its G allele frequency was also significantly higher in the bone metastasis group than that in the no-metastasis group (*P* = 0.019); it could significantly increase the occurrence risk of bone metastasis in breast cancer patients, too (OR = 1.486, 95%CI=1.068-2.068). This result also survived Bonferroni correction for multiple testing (P = 0.019 < 0.025).

### 3.3 The linkage analysis of RANKL polymorphisms in bone metastasis

We also detected the linkage situation of *RANKL* rs7325635 and rs2277438 polymorphisms by Haploview. A strong linkage disequilibrium existed between these two polymorphisms (D′=1.0, r^2^ = 0.321) and three haplotypes G–G, G–A, and A–A were discovered. Compared with the common haplotype, A–A and G–A haplotype frequencies were significantly lower in bone metastasis patients than that in no-metastasis patients, but only A–A haplotype frequency difference was significant (*P* = 0.030); it was significantly related to the decreased risk of bone metastasis development in breast cancer patients (OR = 0.647, 95%CI=0.436-0.959, **Table 3**).

**Table 3:** The roles of haplotypes of *RANKL* polymorphisms in bone metastasis of breast cancer patients.

rs7325635-rs2277438	Haplotype (%)		*P*	OR (95%CI)
	Cases (n=139, %)	Controls (n=152, %)		
G-G	132 (47.48)	115 (37.83)	–	Ref.
G-A	74 (26.62)	92 (30.26)	0.077	0.701 (0.472-1.040)
A-A	72 (25.90)	97 (31.91)	**0.030**	**0.647 (0.436-0.959)**

OR, odds ratio; 95%CI, 95% confidence interval.

Bold values indicate statistical significance (P < 0.05).

### 3.4 The multivariate logistic analysis of clinical indexes and RANKL polymorphisms in bone metastasis occurrence in breast cancer patients

We analyzed the combined effect of effective clinical indexes and *RANKL* polymorphisms in bone metastasis development using multivariate logistic analysis. The results indicated that family history of breast cancer, Ki67, and rs2277438 was positively correlated with the development risk of bone metastasis in breast cancer patients (*P* < 0.05). Moreover, menopausal state, clinical staging, and rs7325635 were negatively correlated with the risk of bone metastasis (*P* < 0.05). The detailed results are shown in **Table 4**.

**Table 4:** Multivariate logistic analysis of clinical features and *RANKL* polymorphisms in bone metastasis occurrence in breast cancer patients.

Index	*P*	OR	95%CI
Family history of breast cancer	**0.029**	2.200	1.082-4.475
Menopausal state	**0.001**	0.416	0.245-0.707
Clinical staging	**0.015**	0.506	0.292-0.877
Molecular subtyping	0.352	0.893	0.704-1.133
Location	0.116	1.530	0.900-2.599
Ki67	**<0.001**	6.728	3.108-14.564
Rs7325635	**0.009**	0.582	0.388-0.872
Rs2277438	**0.031**	1.474	1.037-2.094

OR, odds ratio; 95%CI, 95% confidence interval.

Bold values indicate statistical significance (P < 0.05).

## 4 Discussion

Breast cancer shows metastatic heterogeneity with priority to different organs (22). The bone is considered as the most common distant metastatic site in breast cancer, and the occurrence rate of bone metastasis is as high as 60%-80%. Its emergence greatly affects the quality of life and prognosis of breast cancer patients (23). The study also shows that patients with single bone metastasis have a better prognosis than those with multiple bone metastasis. Moreover, bone metastasis can cause hypercalcemia, pathological fractures, spinal cord compression, intractable pain, etc., which leads to great pain to the patients and even endangers their lives (24). So, it is urgently needed to look for the pathogenesis of bone metastasis in patients with breast cancer.

Bone metastasis of breast cancer is a complex process and affected by numbers of genetic and environmental factors. So far, some factors have been proved to be involved in bone metastasis development. For instance, Ye et al. considered that age, grade, histologic type, surgery of breast lesions, and subtypes were the independent factors for bone metastasis of breast cancer patients (25). Liu and his colleagues also considered that tumor size, breast cancer subtype, American Joint Committee on Cancer (AJCC) T stage, AJCC N stage, age, and marital status were the independent risk factors for bone metastasis of breast cancer patients (26). In the study of Liu et al., positive PR was a protective factor for bone metastasis; positive HER2, lymph node metastasis, non-lobular or ductal breast cancer, and increasing T stage were the risk factors for bone metastasis in breast cancer patients (27). In our study, family history of breast cancer, menopausal state, molecular subtyping, location, and Ki67 were the influenced factors for bone metastasis, but not obesity, pathological type, tumor size, HER2, PR, or ER status. Some different results with previous studies may attribute to different study populations, sample size, statistical approach, and grouping method. The detailed comparison of risk factors across different studies is summarized in **Table 5**. In addition to the above clinical factors and genetic polymorphisms, other factors have also been reported to be involved in bone metastasis of breast cancer. For instance, tumor microenvironment factors such as TGF-β signaling (8), bone remodeling environment (10), and immune suppression (5) have been demonstrated to contribute to bone metastasis development. These factors suggest that bone metastasis is a multifactorial event, and the genetic polymorphisms investigated in the present study represent only one component of the complex pathogenesis.

**Table 5:** Comparison of identified risk factors for bone metastasis in breast cancer across different studies.

Risk factor	Present study	Ye et al. (25)	Liu et al. (26)	Liu et al. (27)
Age	NS	Significant	Significant	–
Family history	Significant	–	–	–
Menopausal state	Significant	–	–	–
Clinical staging	Significant	Significant	Significant	–
Tumor size	NS	–	Significant	Significant
HER2 status	NS	–	–	Significant
PR status	NS	–	–	Significant (protective)
ER status	NS	–	–	–
Molecular subtyping	Significant	Significant	Significant	–
Ki67	Significant	–	–	–
Location (laterality)	Significant	–	–	–

NS, not significant; -, not investigated.

Certainly, genetic factors also play a vital role in bone metastasis occurrence of breast cancer patients. For example, *RUNX2* is deemed to promote breast cancer progression and bone metastasis through recruiting the NuRD(MTA1)/CRL4B complex (28). *TOR1B* serves as an indicator for bone metastasis in breast cancer patients (29). The study of Ferreira et al. indicated that *RANK* rs34945627 polymorphism was a risk factor for bone metastasis in patients with breast cancer (30). The polymorphisms in *ABCC2* and *ABCB1* are independently associated with bone metastasis in breast cancer patients (31). The *RANKL/RANK* system has been proved to be involved in breast cancer bone metastasis (32). Increasing evidence indicated that inhibiting *RANKL* not only resulted in reduced bone metastasis of breast cancer but also has antitumor effects (21). Beyond breast cancer, the RANKL/RANK pathway has also been implicated in the progression and metastasis of other cancer types. RANKL has been reported to be involved in the regulation of tumor immunity, tumor cell migration, and the formation of a bone metastatic microenvironment across multiple solid tumors (19). The clinical application of denosumab, a monoclonal antibody targeting RANKL, for preventing skeletal-related events in patients with bone metastasis from various cancers further supports the broad significance of this pathway (19). These findings suggest that RANKL polymorphisms may have implications beyond breast cancer and deserve investigation in other cancer types as well. Nowadays, many single-nucleotide polymorphisms (SNPs) have been discovered and studied in some diseases. Rs2277438 and rs7325635 are two of the most common SNPs in RANKL, both located in the intronic region. Rs2277438 is situated in intron 1 of the TNFSF11 gene, close to the intron 1/exon 2 boundary. Although it is a non-coding variant, previous studies have shown that carriers of the rs2277438 AG genotype exhibited significantly elevated serum RANKL levels compared to homozygous genotypes in rheumatoid arthritis patients, suggesting a potential regulatory effect on RANKL expression. Furthermore, a recent study identified an osteocytic cell-specific intronic enhancer in the TNFSF11 gene locus enriched for the active enhancer marker H3K27ac, indicating that intronic regions of TNFSF11 may harbor functional regulatory elements. However, whether rs2277438 directly disrupts any regulatory element or transcription factor binding site has not been experimentally validated to date. Rs7325635 is reported to be significantly correlated with hip bone loss in postmenopausal women (33). Rs2277438 also presents a significant correlation with bone disease (34). Moreover, the linkage disequilibrium is discovered between the two.

Therefore, in the current study, we investigated the role of *RANKL* rs2277438 and rs7325635 polymorphisms in the risk of bone metastasis among patients with breast cancer. The results indicated that the GG genotype and G allele of rs2277438 were significantly correlated with the increased risk of bone metastasis. So, rs2277438 may be an effective indicator for the risk of breast cancer bone metastasis in the Chinese Han population. However, we could not find the significant frequency difference in genotypes or alleles of rs7325635 between bone metastasis patients and no-metastasis patients, so rs7325635 was not independently correlated with bone metastasis of breast cancer patients. In a previous study, the *RANKL* rs9533156 C allele has been considered as a protective factor for breast cancer bone metastasis (35). Therefore, the study of *RANKL* polymorphisms may be an important issue for identifying bone metastasis pathogenesis in patients with breast cancer. It should be noted that these RANKL polymorphisms exist in the general population regardless of breast cancer status. rs2277438 and rs7325635 have been previously associated with bone metabolism phenotypes in non-cancer populations, such as osteoporosis and bone mineral density variations (33, 34). Therefore, these polymorphisms may represent inherent genetic susceptibility factors for altered bone remodeling, which could independently predispose individuals to bone-related complications. In breast cancer patients, such preexisting genetic backgrounds may further amplify the risk of bone metastasis when combined with tumor-derived factors. Moreover, the linkage analysis showed that the A–A haplotype played a protective role for bone metastasis occurrence in our population, so the linkage disequilibrium pattern and haplotype distribution of RANKL polymorphisms deserve attention in bone metastasis research. It should be clarified that the haplotype analysis in this study reflects linkage disequilibrium between the two SNPs rather than biological epistasis or functional interaction. Whether these SNPs exert a true biological synergistic effect on bone metastasis requires further functional studies. Additionally, a multivariate logistic analysis also found that bone metastasis in breast cancer patients was positively related to family history of breast cancer, Ki67, and rs2277438, but a negative association with menopausal state, clinical staging, and rs7325635.

Several shortcomings need to be stated in this study. The relatively small sample size and only one Chinese Han population in this study may reduce the accuracy of our study results. Due to the limited sample size, we were unable to perform subgroup analysis stratified by molecular subtypes (luminal A, luminal B, HER2 overexpression, and TNBC) to determine whether specific RANKL polymorphisms are preferentially associated with bone metastasis in particular breast cancer subtypes. This is an important issue that warrants future investigation with larger cohorts. The mechanism of RANKL polymorphisms in bone metastasis among breast cancer patients is not disclosed in our study. So, more studies are needed to prove the current results and uncover the possible mechanism in bone metastasis development using a well-designed, large sample size and more populations. Future studies should also explore the association between RANKL polymorphisms and bone metastasis risk stratified by breast cancer molecular subtypes.

## 5 Conclusion

The *RANKL* rs2277438 variant may contribute to the risk of bone metastasis development among breast cancer patients, but not rs7325635. Moreover, the haplotype analysis suggests that the combined allelic pattern of these two polymorphisms is associated with bone metastasis risk. We also discover that bone metastasis of breast cancer patients is positively related to family history of breast cancer, Ki67, and rs2277438 but negatively associated with menopausal state, clinical staging, and rs7325635 based on a multivariate logistic analysis.

## Data Availability

The raw data supporting the conclusions of this article will be made available by the authors, without undue reservation.
